# Single- and Multi-Trait GWASs Combined with Genetic Parameter Estimation Reveal Candidate Genes for Body Conformation Traits in Sika Deer (*Cervus nippon*)

**DOI:** 10.3390/ani16091325

**Published:** 2026-04-27

**Authors:** Hexuan Gao, Tianjiao Wang, Ranran Zhang, Xu Chen, Huanhuan Fan, Sukun Yang, Shiwu Dong, Handa Zhang, Lixin Tang, Xiumei Xing

**Affiliations:** Key Laboratory of Genetics, Breeding and Reproduction of Special Economic Animals, Ministry of Agriculture and Rural Affairs, Institute of Special Animal and Plant Sciences, Chinese Academy of Agricultural Sciences, Changchun 130112, China; 15541833897@163.com (H.G.); wangtianjiao@caas.cn (T.W.); heavenranran@163.com (R.Z.); 13354596250@163.com (X.C.); fanhuanhuan911@163.com (H.F.); 15634530001@163.com (S.Y.); dongshiwu2017@126.com (S.D.); zhd1113zhd@163.com (H.Z.); tanglixin1217@163.com (L.T.)

**Keywords:** sika deer, body conformation traits, genetic parameters, single-trait GWAS, multi-trait GWAS

## Abstract

Both the productive efficiency and physical health of sika deer are strongly linked to their body conformation phenotypes. Breeding sika deer with excellent growth traits using molecular breeding technologies has become essential. Phenotypic data of 12 body conformation traits were measured for 613 sika deer across three age groups, including body traits and cephalic traits. Genetic typing was performed using the Sika Deer 100K SNP Liquid Chip, and genetic parameters were estimated through animal models to obtain the heritability and genetic correlation of traits within each age group. Single-trait and multi-trait GWASs were conducted, using GEMMA software to identify gene variants significantly associated with sika deer body conformation traits. Most of the 12 body conformation traits exhibited moderate to high heritability. The single-trait GWAS identified 49 SNPs (38 candidate genes), while multi-trait GWAS detected 134 SNPs (80 candidate genes), including 114 novel loci. A total of 163 SNPs and 196 candidate genes were identified, with 17 genes detected by both methods. These genes may be involved in pain perception, cell cycle regulation, immune response, and protein ubiquitination. Two significant KEGG pathways were enriched: steroid hormone biosynthesis and drug metabolism–cytochrome P450. Collectively, these detected loci and genes may serve as potential genetic resources for marker-assisted breeding, contributing to subsequent genetic improvement of body conformation in sika deer.

## Simple Summary

Both the productive efficiency and physical health of sika deer are strongly linked to their body conformation phenotypes. We aimed to explore the genetic parameters of body conformation traits in sika deer and identify related gene variations, providing a reference for breeding. We collected body measurements for 12 conformation traits from 613 sika deer across three age groups. Using the 100K SNP Liquid Chip, we estimated heritability and genetic correlations for these traits and performed single-trait and multi-trait genome-wide association studies to detect key genetic markers. Most of the 12 body conformation traits exhibited moderate to high heritability, indicating substantial genetic potential for improving body conformation in sika deer. A panel of 163 SNP sites was screened, with 196 relevant candidate genes functionally annotated. Among them, 17 shared genes were captured synchronously by both GWAS analytical pipelines. Multiple biological regulatory pathways, screened via GO and KEGG enrichment, are likely modulated by these candidate genes. Two significantly enriched KEGG pathways were identified: steroid hormone biosynthesis and drug metabolism–cytochrome P450. Collectively, these detected loci and genes may serve as potential genetic resources for marker-assisted breeding, contributing to subsequent genetic improvements of body conformation in sika deer.

## 1. Introduction

Sika deer (*Cervus nippon*), an important special economic animal in East Asia, is renowned as a precious species whose “every part holds value.” This reputation derives from the high medicinal, health-promoting, and economic values of its products, particularly velvet antlers and venison. China is the world’s largest producer and consumer of sika deer, and the sika deer industry continues to expand. In particular, in 2020, sika deer were included in the National Catalog of Livestock and Poultry Genetic Resources Breeds [1], which indicates that the sika deer farming industry has great prospects.

However, sika deer breeding has long focused on the economic trait of velvet antlers, prioritizing antlers over venison and neglecting the development of meat-type lines. Being rich in protein, low in fat, and highly nutritious, venison has the potential to enjoy a large domestic market, yet no specialized meat-type sika deer breed exists [2]. Current genetic gains have been achieved mainly through phenotypic selection for velvet antler weight, but this strategy is constrained by long generation intervals and a low genetic progress rate. There is therefore an urgent need to dissect the genetic architecture of economically important traits, thereby accelerating the application of genomic breeding techniques.

In animal husbandry, body conformation serves as a key phenotypic indicator for evaluating individual growth performance. To date, most studies on sika deer have focused on antler traits, while investigations into body conformation traits remain limited. As an important suite of economic traits, body conformation may directly influence antler yield [3], slaughter performance [4], and fertility [5,6,7,8] and can serve as a core breeding objective. Moreover, selection for improved body conformation can accelerate the genetic progress of other economic traits. Therefore, a systematic investigation of body conformation traits represents a critical component of sika deer breeding programs. Genome-wide association analyses (GWAS) are increasingly applied to the genetic dissection of complex traits. Existing studies have used the GWAS method to explore the genetic structure of body conformation traits in Cervidae [9,10]. The integration of SNP chips with GWAS enables the identification of molecular markers associated with economically important phenotypes. The availability of a dedicated 100K SNP Liquid Chip for sika deer breeding provides valuable support for conducting GWAS on these traits.

Most GWASs for sika deer body conformation traits have focused on single-trait analysis, which ignores phenotypic correlations caused by pleiotropy and linkage disequilibrium. Multi-trait GWAS, by contrast, improves detection accuracy by integrating multiple traits and reducing multiple-testing bias [11,12]. Given the genetic correlations among body traits [13,14,15], this strategy is more suitable for exploring the genetic architecture of sika deer. A previous study in cattle also detected numerous significant SNPs and candidate genes using single- and multi-trait GWAS [16]. Another group [17] used the Sheep 40K Liquid Chip to perform single-trait and multi-trait GWASs on 799 Hulunbuir sheep for body conformation traits, with the identification of three significant SNPs and three candidate genes associated with body conformation traits.

This study focused on 12 body conformation traits in sika deer across three distinct age groups. We hypothesized that significant SNPs and candidate genes associated with body conformation traits can be identified by single-trait and multi-trait GWAS. We aimed to collect basic phenotypic data for these traits and genomic data and perform a series of analyses: genetic parameter estimation, single-trait GWAS, multi-trait GWAS, and gene enrichment analysis. The identified SNPs and candidate genes will provide reliable molecular markers for genomic selection, supporting molecular breeding programs aimed at improving body conformation and establishing a genetic basis for meat-type sika deer improvement.

## 2. Materials and Methods

### 2.1. Experimental Animals

**Figure 1:**
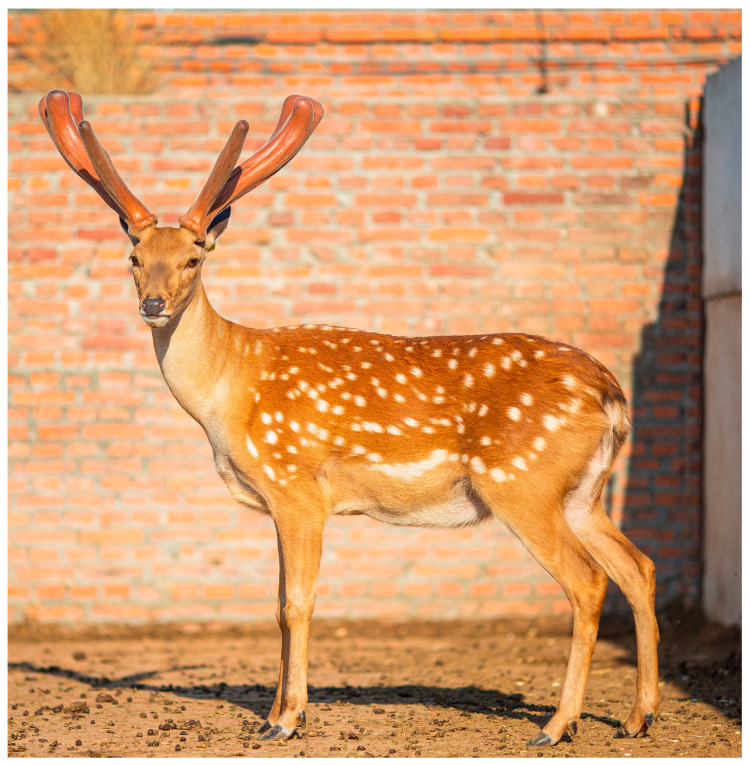
Photo of an adult male sika deer. (Photo by the authors).

A total of 613 healthy male sika deer were used in this study. All sika deer (Figure 1) were sourced from three standardized commercial farms in Jilin Province, China, and raised under consistent feeding and management practices, including a unified diet, feeding regime, and rearing environment. All individuals were clinically healthy and verified to be free of observable diseases.

To account for age effects, deer were divided into three age groups: Group 1: 2-year-old individuals (*n* = 254); Group 2: 3-year-old individuals (*n* = 189); Group 3: individuals aged 4 years and older (*n* = 322).

All experimental procedures involving animals were approved by the Animal Management and Welfare Ethics Committee of the Institute of Special Animal and Plant Sciences, Chinese Academy of Agricultural Sciences (approval No. ISAPSAEC-2024-034). All methods were performed in accordance with the ARRIVE guidelines.

### 2.2. Phenotypic Data Collection

Twelve body conformation traits were measured for each deer by two trained technicians following the standardized measurement protocol for livestock genetic resources [18]. All measurements were conducted in the morning after 12 h of fasting with free access to water, and completed before velvet antler harvesting to avoid potential interference. Each trait was measured three consecutive times, and the mean value was used as the final phenotypic record to ensure accuracy.

Measured traits were classified into two categories:

Body traits:

- Body Weight (BW): Fasting body weight before antler collection (kg)
- Body Height (BH): Vertical distance from the highest point of the withers to the ground (cm)
- Body Length (BL): Straight-line distance from the anterior edge of the shoulder joint to the posterior edge of the hip joint (cm)
- Chest Circumference (CC): Chest circumference measured posterior to the scapula (cm)
- Chest Depth (CD): Straight-line distance from the withers to the sternum (cm)
- Tube Circumference (TC): Circumference of the left forelimb at the upper one-third of the cannon bone (cm)
- Tail Length (TL): Straight-line distance from the tail base to the tail tip (cm)

Cephalic traits:

- Head Length (HL): Straight-line distance from the top of the forehead to the upper edge of the nasal mirror (cm)
- Forehead Width (FW): Maximum forehead width, measured between the lateral edges of the two orbits (cm)
- Horn Handle Distance (HHD): Straight-line distance between the centers of the left and right horn handles (cm)
- Left/Right Horn Handle Girth (HHGL/HHGR): Circumference of the middle portion of the left and right horn handles, respectively (cm)

Levene’s test was used to assess the homogeneity of variances across age groups for all measured traits. The results indicated that variance homogeneity was not uniformly satisfied across age groups for several traits. Since GWAS was performed separately within each age group, the between-group variance heterogeneity did not influence the association analyses.

### 2.3. Genotype Data and Quality Control

In this study, 613 sika deer were selected for jugular vein blood collection, and genomic DNA was extracted from blood samples of these deer via the Vazyme Blood Genome Extraction Kit (Vazyme Biotech Co., Nanjing, China). Five mL of venous blood was obtained from each experimental sika deer and subsequently stored under −80 °C freezing conditions. Genotyping was performed via the Sika Deer 100K SNP Liquid Chip, which initially contained 100,000 SNP markers.

The raw SNP data was subjected to systematic quality control (QC) utilizing PLINK v1.90 [19], following strict exclusion criteria to ensure data reliability: (1) SNPs with a minor allele frequency (MAF) < 0.05; (2) SNPs with a missing rate (MISS) > 0.1; (3) SNPs with a Hardy–Weinberg equilibrium (HWE) *p*-value < 1.0 × 10^−4^. After QC, a total of 87,182 high-quality SNP markers were retained. LD pruning was performed using PLINK with the parameter—indep-pairwise 50 5 0.2, resulting in 86,996 independent SNPs. The 86,996 independent SNPs were used for multiple testing correction to ensure a reasonable genome-wide significance threshold. No further SNPs were added or excluded during subsequent analyses, maintaining consistency across the study. These high-quality SNP markers were approximately evenly distributed across the chromosomes, which was verified by visualization (Figure S1), and were suitable for subsequent genome-wide association study (GWAS) of sika deer.

### 2.4. Estimation of Genetic Parameters for Body Conformation Traits

Descriptive statistics for the twelve body conformation traits—including maximum, minimum, mean, variance, standard deviation, and coefficient of variation—were calculated. Prior to this, phenotypic data preprocessing was performed in R software (version 4.3.0). Outliers were removed based on the 3σ rule. Normality was examined using the Shapiro–Wilk test. Histograms were plotted to visually inspect the distribution of each phenotypic trait. Genetic parameters (heritability, genetic correlation, and phenotypic correlation) for these traits were estimated using a linear mixed model fitted by the asreml() function in the ASReml-R package (version 4.2.0.276), followed by parameter extraction using the vpredict() function. This package is designed for fitting linear mixed models via residual maximum likelihood (REML), a method particularly suited to genetic parameter estimation in plant and animal breeding due to its unbiased estimation of variance components even when data are unbalanced.

### 2.5. Animal Model

Single-trait and two-trait genomic best linear unbiased prediction (GBLUP) models were used to estimate the variance components of 12 body conformation traits, and the genetic parameters (heritability, genetic correlation, and phenotypic correlation) were further calculated based on the estimated variance components.

All genetic analyses were based on a unified linear mixed model (LMM) framework with shared random effects structure. The general model is defined as follows:

(1) y=μ+Xf+Zg+e,

where y is the *n* × 1 vector of phenotypic records (*n* = number of individuals); μ is the overall mean; f is the p × 1 vector of fixed effects, including deer farm effect (categorical) and population stratification effect (first three principal components derived from SNP genotypes); g is the *n* × 1 vector of random polygenic effects, following g**∼ N**(**0,** $G\sigma_{g}^{2}$), where G is the n × n genomic relationship matrix constructed from SNP markers using the VanRaden method; e is the *n* × 1 vector of residual effects, following e**∼ N**(**0,** $I\sigma_{e}^{2}$); and X (*n* × *p*) and Z (*n* × *n*) are the incidence matrices corresponding to fixed and random effects, respectively.

#### 2.5.1. Variance Component Estimation (GBLUP)

To estimate variance components and genetic parameters, we applied single-trait and two-trait GBLUP models based on the general LMM framework (Equation (1)). The single-trait GBLUP model (Model 1) follows Equation (1), where the terms are as defined in Equation (1), with g being the vector of genomic additive genetic effects, following g**∼**N (**0, *G***$\sigma_{g}^{2}$).

The two-trait GBLUP model (Model 2) extended this to bivariate analysis:

(2) $$y=\left[\begin{array}{c} y_{1}\\ y_{2} \end{array}\right],\;f=\left[\begin{array}{c} f_{1}\\ f_{2} \end{array}\right],\;g=\left[\begin{array}{c} g_{1}\\ g_{2} \end{array}\right],\;e=\left[\begin{array}{c} e_{1}\\ e_{2} \end{array}\right],$$

where subscripts 1 and 2 denote the two traits. The variance-covariance matrices were specified as $Var\left(g\right)=G0⊗G$ and $Var\left(e\right)=R0⊗I$, where G0 (2 × 2) and R0 (2 × 2) are the genomic genetic and residual (co)variance matrices between traits, respectively, and ⊗ denotes the Kronecker product.

The narrow-sense heritability ($h^{2}$) was calculated as:

(3) $$h^{2}=\frac{\sigma_{g}^{2}}{\sigma_{g}^{2}\;+\;\sigma_{e}^{2}},$$

Genetic correlations ($r_{G}$) and phenotypic correlations ($r_{P}$) between pairs of traits were calculated as:

(4) $$r_{G}=\frac{{Cov}_{G_{XY}}}{\sqrt{\sigma_{G_{X}}^{2}\sigma_{G_{Y}}^{2}}},$$

(5) $$r_{P}=\frac{{Cov}_{P_{XY}}}{\sqrt{\sigma_{P_{X}}^{2}\sigma_{P_{Y}}^{2}}},$$

where ${Cov}_{G_{XY}}$ is the genomic additive genetic covariance (from G0 in Model 2), $\sigma_{G_{X}}^{2}$ and $\sigma_{G_{Y}}^{2}$ are the genomic additive genetic variances (diagonal elements of G0), and ${Cov}_{P_{XY}}$ = ${Cov}_{G_{XY}}$ + ${Cov}_{e_{XY}}$ (where ${Cov}_{e_{XY}}$ is from R0), and $\sigma_{P_{X}}^{2}$= $\sigma_{G_{X}}^{2}$+$\sigma_{e_{X}}^{2}$, $\sigma_{P_{Y}}^{2}$ = $\sigma_{G_{Y}}^{2}$+$\sigma_{e_{Y}}^{2}$.

#### 2.5.2. Genome-Wide Association Analysis (GWAS)

GWAS was performed using the same LMM framework as described in Section 2.5, with the addition of the tested SNP as a fixed effect. For single-trait GWAS, the model for testing marker k was:

(6) $$y=\mu +Xf+w_{k}\beta_{k}+Zg+e,$$

where $w_{k}$ is the genotype vector for SNP k, $\beta_{k}$ is its allele substitution effect (fixed), and the random effects g**∼** N (**0,** $G\sigma_{g}^{2}$) and e**∼** N (**0,** $I\sigma_{e}^{2}$) have the same covariance structure as in Equation (1).

Multi-trait GWAS (MT-GWAS) extended this to multivariate analysis:

(7) $$Y=\mu +Xf+w_{k}\beta_{k}+Zg+E,$$

where Y is the *n* × *d* phenotypic matrix (*d* = number of traits), $\beta_{k}$ is the *d* × 1 vector of SNP effects across traits, and the random effects follow Var (g) **= *V_g_***⊗ **G** and Var (E) **= *V_e_***⊗ **I_n_**, with variance-covariance structures ***V_g_*** and ***V_e_*** (*d* × *d*) analogous to G0 and R0 in the two-trait GBLUP model (Model 2).

Independent single nucleotide polymorphisms (SNPs) were estimated using PLINK software (v1.9). A genome-wide significance threshold was established via the Bonferroni correction method, defined as *p* < 0.05/N, where N is the number of independent SNPs. For our analysis, this threshold was calculated as *p* < 5.75 × 10^−7^ = 0.05/86,996). In addition, associations reaching *p* < 1.0 × 10^−5^ within a specific chromosomal region were considered suggestive and were also retained for candidate gene investigation, following established practice [20]. Finally, Manhattan and quantile–quantile (Q–Q) plots were generated using the CMplot function in R.

### 2.6. Population Genetics Analysis

GEMMA software (version 0.98.3) was used to construct the genomic relationship matrix G to account for genetic relatedness among individuals in the sika deer population. The result was visualized using the ‘ggplot2’ package in R. In addition, a principal component analysis (PCA) was performed using all SNPs. The first two PCs were visualized using the R package ‘ggplot2’.

### 2.7. Gene Annotation and Functional Enrichment Analysis

The SNP position information in the Sika Deer 100K SNP Liquid Chip was based on the sika deer reference genome (version MHL_v1.0) [21]. We performed linkage disequilibrium (LD) decay analysis to characterize genome-wide LD patterns in our sika deer population. The LD analysis revealed that rapid LD decay occurred within a short physical distance in our population (Figure S3). LD between SNP markers was calculated using the jar package Haploview (version: 4.2, [22]). To comprehensively capture both strongly linked coding regions and potential functional genes that may affect trait variation, significant SNPs within a 50-kb upstream and downstream range were annotated via ANNOVAR software (version 2018-04-16) [23]. This window size is widely adopted in GWAS of ruminant species [16,24,25,26]. Functional enrichment analysis, including Gene Ontology (GO) and KEGG pathway enrichment, was performed using the clusterProfiler package in R software (version 4.17.0). The Benjamini–Hochberg false discovery rate (FDR) method was applied to correct for multiple testing, and terms with an adjusted *p*-value < 0.05 were considered significantly enriched.

## 3. Results

### 3.1. Descriptive Statistical Analysis of the Phenotypic Data

Descriptive statistical analysis was performed on the mean, median, maximum, minimum, variance, standard deviation (SD), and coefficient of variation (CV) of the 12 body conformation traits of 613 sika deer (Table 1). Based on Shapiro–Wilk normality tests and frequency distribution histograms with density curves (Figure S2), all body conformation traits were approximately normally distributed.

### 3.2. Population Structure Analysis

**Figure 2:**
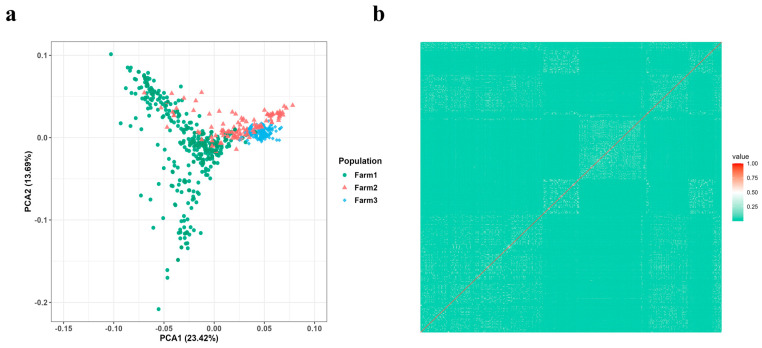
The structure analysis of the sika deer population (**a**) PCA result of population stratification of sika deer. (**b**) Genetic relationships between sika deer individuals.

Before performing GWAS, in order to prevent false positives and determine the model, the population structure of the tested deer should be analyzed and appropriately corrected. We used PLINK software to calculate the percentage of variance explained by the first ten principal components (PCs), and selected the first three principal components for further analysis. PCA indicated slight population stratification (Figure 2a). PC1 and PC2 accounted for 20.71% and 13.80% of the total variance, respectively. Therefore, we selected the first three PCs as covariates and included them in the GWAS model. The correlation coefficients for all individual pairs ranged from 0 to 0.250, indicating no genetic relatedness among the samples (Figure 2b).

### 3.3. Estimation of Heritability and Trait Correlation

**Figure 3:**
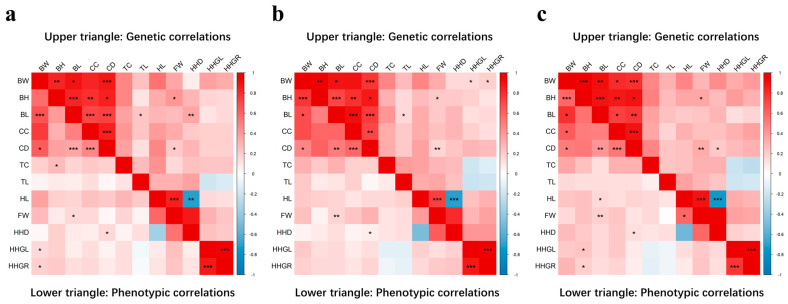
Genetic correlations between conformation traits in sika deer. The upper triangle represents genetic correlation, while the lower one represents phenotypic correlation. Red represents a positive correlation and blue represents a negative correlation. *: significant level less than 0.05, **: significant level less than 0.01, ***: significant level less than 0.001. (**a**) 2-year-old sika deer. (**b**) 3-year-old sika deer. (**c**) ≥4-year-old sika deer.

We estimated the genetic parameters of 12 body conformation traits of sika deer across three age groups (2 years, 3 years, and ≥4 years), with the results shown in Table 2 and Figure 3. Specifically, the heritability of body traits in 2-year-old sika deer ranged from 0.24 (TL) to 0.54 (CC), and the heritability of cephalic traits ranged from 0.16 (FW) to 0.36 (HL). The heritability of body traits in 3-year-old sika deer ranged from 0.24 (TL) to 0.51 (BL), and the heritability of cephalic traits ranged from 0.18 (FW) to 0.33 (HL). The heritability of body traits in sika deer aged ≥4 years ranged from 0.25 (TL) to 0.47 (BW), and the heritability of cephalic traits ranged from 0.13 (HHD) to 0.37 (HHGR).

Most of the 12 body conformation traits exhibited moderate to high heritability. For 2-year-old sika deer, FW showed low heritability (0.16). The heritability values of BW, BL, TL, HL, HHD, HHGL and HHGR were moderate (0.21–0.38), and those of BH, CC, CD and TC were high (0.43–0.54). For 3-year-olds, the heritability of FW remained low (0.18). The heritability values of BH, CD, TL, HL, HHD, HHGL and HHGR were moderate (0.21–0.38), and those of BW, BL, CC and TC were high (0.41–0.51). For ≥4-year-olds, the heritability of HHD was low (0.13), the heritability values of BH, CC, CD, TL, HL, FW, HHGL and HHGR were moderate (0.25–0.37), and those of BW, BL and TC were high (0.44–0.47).

Among 2-year-old sika deer, the genetic correlations of body traits ranged from 0.11 to 0.88, all being positive correlations. In particular, BW, BH, BL, CC, and CD exhibited very high genetic correlations, while TC and TL showed moderate to low genetic correlations with other traits. The genetic correlations of cephalic traits ranged from −0.67 to 0.95, with HL-HHD (−0.67) showing negative genetic correlation and the others showing positive genetic correlations. Specifically, HL-FW (0.85), HL-HHD (−0.67), FW-HHD (0.87), and HHGL-HHGR (0.95) had high genetic correlations, while the remaining traits exhibited moderate to low genetic correlations.

Among 3-year-old sika deer, the genetic correlations of body traits ranged from 0.15 to 0.94, all being positive correlations. In particular, BW, BH, BL, CC, and CD exhibited very high genetic correlations, while TC and TL showed moderate to low genetic correlations with other traits. The genetic correlations of cephalic traits ranged from −0.73 to 0.94, with HL-HHD (−0.73) showing negative genetic correlation and the others showing positive genetic correlations. HL-FW (0.73), HL-HHD (−0.73), FW-HHD (0.81), and HHGL-HHGR (0.94) had high genetic correlations, while the remaining traits exhibited moderate to low genetic correlations.

Among ≥4-year-old sika deer, the genetic correlations of body traits ranged from 0.17 to 0.95, all being positive correlations. In particular, BW, BH, BL, CC, and CD exhibited very high genetic correlations, while TC and TL showed moderate to low genetic correlations with other traits. The genetic correlations of cephalic traits ranged from −0.74 to 0.97, with HL-HHD (−0.74) showing negative genetic correlation and the others showing positive genetic correlations. HL-FW (0.89), HL-HHD (−0.74), FW-HHD (0.95), and HHGL-HHGR (0.97) had high genetic correlations, while the remaining traits exhibited moderate to low genetic correlations.

### 3.4. Single-Trait and Multi-Trait GWASs

#### 3.4.1. Assessment of Genomic Inflation Factors (λ)

Genomic inflation factors (λ) were computed for all single-trait and multi-trait GWAS (Table S2). For single-trait analyses, the λ values ranged from 0.972 to 1.048 across age groups, confirming no genomic inflation. For multi-trait analyses, the λ values ranged from 0.941 to 1.238, with most values below 1.15, indicating that population stratification was adequately controlled in all GWAS models.

#### 3.4.2. Overall Analysis Across Ages

To dissect the dynamic genetic architecture underlying the growth traits across different developmental stages, we conducted separate GWAS analyses for three age groups (2, 3, and ≥4 years old) using both single-trait and multi-trait models. This stratified approach was employed to account for the age-dependent heterogeneity in genetic effects, which would likely be masked in a combined overall analysis.

**Figure 4:**
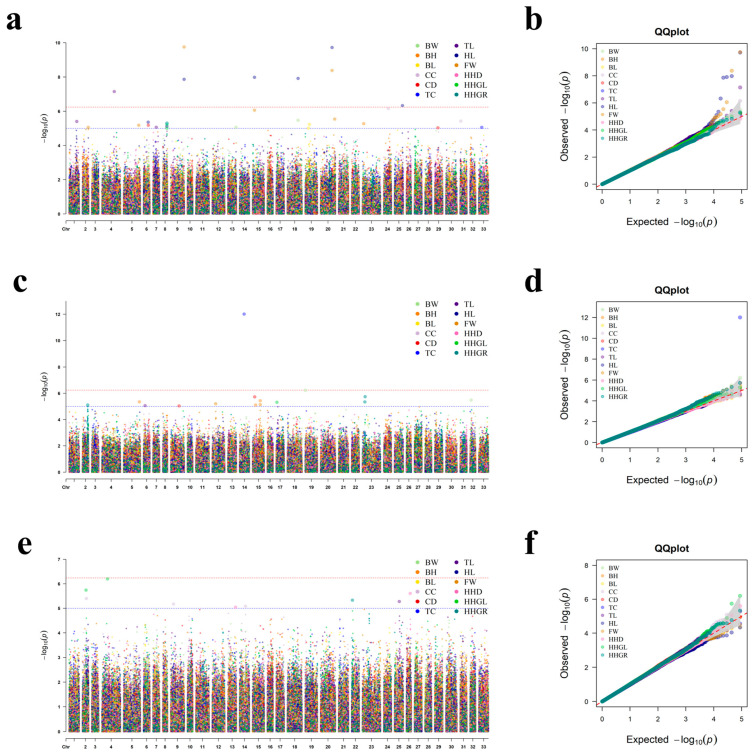
Manhattan and QQ plots for single-trait GWAS. Figures (**a**,**c**,**e**) represent Manhattan plots for 2-year-old sika deer, 3-year-old sika deer, and ≥4-year-old sika deer, respectively. Figures (**b**,**d**,**f**) represent the QQ plots for 2-year-old sika deer, 3-year-old sika deer, and ≥4-year-old sika deer, respectively. The horizontal red dashed line corresponds to the genome-wide significance threshold (*p* < 5.75 × 10^−7^), and the horizontal blue dashed line represents the chromosome-wide suggestive significance threshold (*p* < 1.0 × 10^−5^).

**Figure 5:**
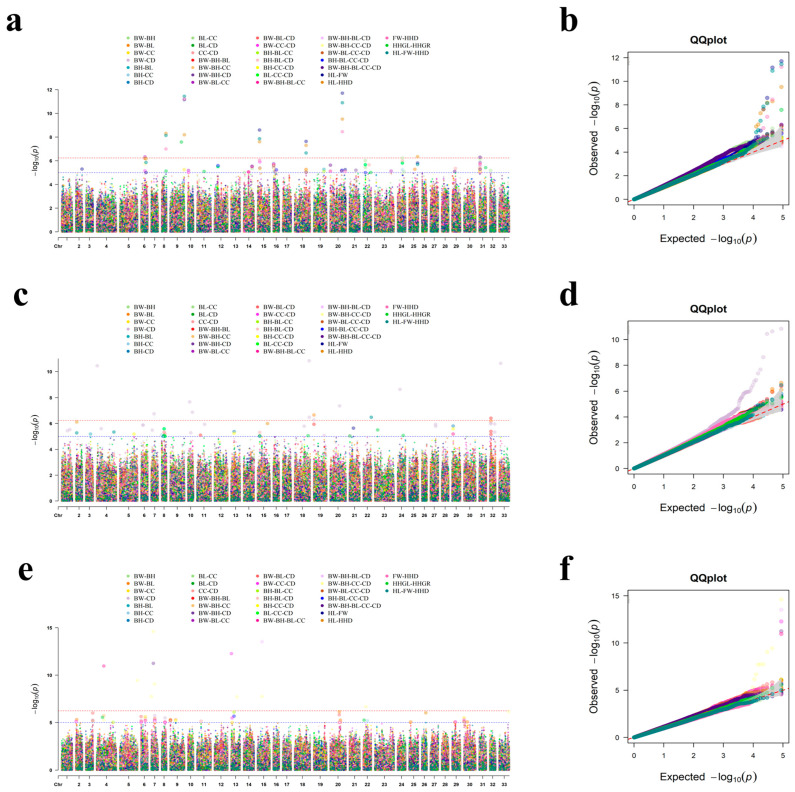
Manhattan and QQ plots for the multi-trait GWAS. Figures (**a**,**c**,**e**) represent Manhattan plots for 2-year-old sika deer, 3-year-old sika deer, and ≥4-year-old sika deer, respectively. Figures (**b**,**d**,**f**) represent the QQ plots for 2-year-old sika deer, 3-year-old sika deer, and ≥4-year-old sika deer, respectively. The horizontal red dashed line corresponds to the genome-wide significance threshold (*p* < 5.75 × 10^−7^), and the horizontal blue dashed line represents the chromosome-wide suggestive significance threshold (*p* < 1.0 × 10^−5^).

In single-trait GWAS for 12 conformation traits across three age groups, we identified a total of 49 significant loci (including four shared across traits). In contrast, multi-trait GWAS detected 134 significant SNPs in total, identifying 113 novel SNPs that were not found in the single-trait analysis. The corresponding QQ plots and Manhattan plots are shown in Figure 4 and Figure 5.

#### 3.4.3. Age-Specific Analysis

Single-trait GWAS identified 25, 15, and 9 significant SNPs (annotated to 22, 24, and 15 genes) in the 2-year-old, 3-year-old, and ≥4-year-old groups, respectively, showing a declining trend with age (Table S3). This suggests that genetic factors explain more phenotypic variance during early growth stages when developmental rates are highest. Conversely, multi-trait GWAS identified 48, 52, and 36 significant SNPs (annotated to 40, 63, and 59 genes) across the three age groups, with the peak number of loci and genes observed in the 3-year-old group. The multi-trait model consistently detected more significant variants and annotated more candidate genes than the single-trait model in all age groups, demonstrating its superior power to identify pleiotropic genetic effects underlying correlated conformation traits across different developmental stages.

#### 3.4.4. Haplotype Block Analysis for Key Conformation Traits

**Figure 6:**
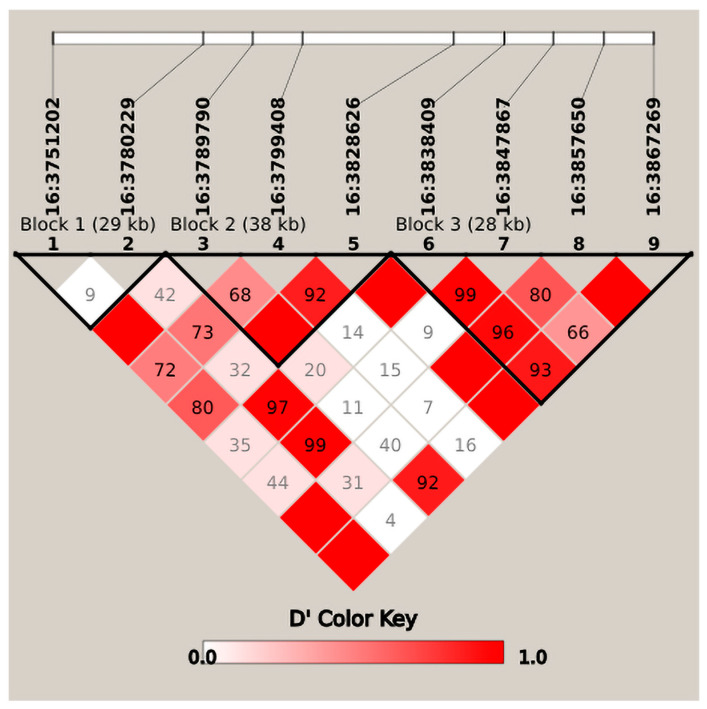
Haplotypes in the 3.78–3.85 Mb region of chromosome 16. The black boxes outline the haplotype blocks, which were defined using the confidence interval method based on pairwise linkage disequilibrium (D′) values.

Additionally, multi-trait genome-wide association scanning revealed a candidate interval on chromosome 16 spanning 3.78–3.85 Mb. Within this interval, two significant SNPs were found to be linked to BW, BH, and CD traits. Moreover, three distinct haplotype blocks, consisting of 2, 3, and 4 SNPs, respectively, were detected in this genomic region. (Figure 6).

### 3.5. Functional Enrichment Analysis of GO and KEGG

**Figure 7:**
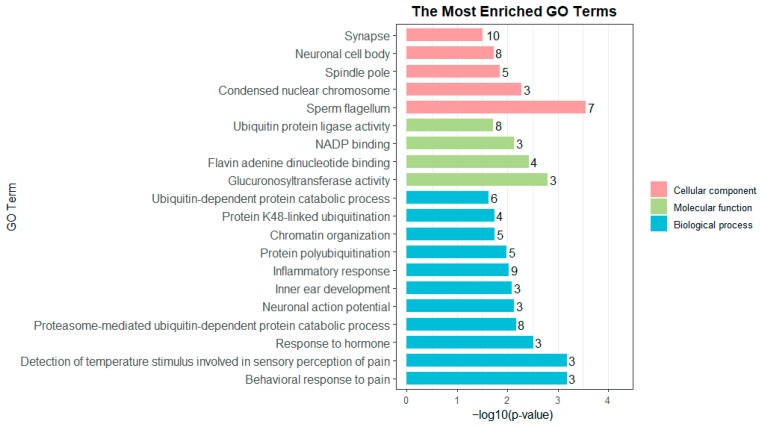
GO analysis of candidate genes for body conformation traits of sika deer.

After removing duplicates where the same SNP or gene was identified in multiple age groups or by both analytical methods, we established a final list of 162 unique significant SNPs with their corresponding proportion of phenotypic variance explained (PVE) and 196 unique candidate genes (Table S1). Among these, 17 genes were identified by both GWASs, including *ZFYVE9*, *CABCOCO1*, *POLA1*, *IGF2R*, *SLC22A1*, *AGAP1*, *GRM8*, *UBE2C*, *MAP3K7CL*, *TCF20*, *CYP2D14*, *ATP6V1G1*, *NDUFA6*, *NYAP2*, *CUL3*, *KCNQ1*, and *SH3GL2*. Functional GO enrichment analysis indicated remarkable enriched terms under the threshold of adjusted *p* < 0.05. In total, 72 functional terms were screened, containing 48 biological process entries, 13 cellular component entries, and 11 molecular function entries. Subsequently, we selected the top 20 GO terms based on adjusted *p*-value for visualization (Figure 7). These terms can mainly be classified into the following categories of biological processes: pain perception and neural signal transduction, cell cycle regulation, immune and inflammatory responses, and protein ubiquitination degradation pathways. The KEGG enrichment results revealed pathways potentially involved in hormone synthesis and metabolic balance, including steroid hormone biosynthesis (*UGT2B4*, *UGT2B31*, *CYP21*, *DD3*; *P. adjust* = 0.0468) and drug metabolism—cytochrome P450 (*UGT2B4*, *UGT2B31*, *FMO1*, *FMO4*; *P. adjust* = 0.0468). Among them, *UGT2B4* and *UGT2B31* were enriched in two pathways (Table 3 and Figure S4).

## 4. Discussion

### 4.1. Estimation of Genetic Parameters for Body Conformation Traits of Sika Deer

To clarify the genetic relationships among 12 body conformation traits of sika deer, we estimated genetic parameters and analyzed the genetic and phenotypic correlations between traits. Growth traits in sika deer may be influenced by both genetic and environmental factors, with heritability serving as the key indicator of genetic contribution. Notably, studies in long-lived, age-structured wild populations have shown that selective pressures and gene expression patterns can differ significantly between age groups [27]. For example, the heritability of body weight rises with age in white-tailed deer [28], and antler-size genetic variance triples from prime to senescent red deer stags [29]. These findings underscore the need for age-stratified analyses to avoid biased estimates and GWAS signals. We therefore took age structure into consideration when estimating heritability and conducting GWAS in sika deer. To the best of our knowledge, we systematically analyzed the differences in genetic parameters across age groups in sika deer for the first time, providing a theoretical basis for age-specific breeding strategies.

According to our analysis, there were little differences in heritability of most traits among the three age groups. This age-conservative heritability suggests that these traits are ideal candidates for marker-assisted selection (MAS), because genetic improvement measures for these traits can be applied consistently across sika deer populations of different ages. In contrast, traits such as body length and chest circumference exhibit significant age-specific differences in heritability. This not only reveals the stage-specific genetic regulation of sika deer body conformation traits but also provides a theoretical basis for accurately identifying age-specific genetic loci while offering insights into the dynamic changes in genetic effects. This refined strategy could significantly enhance the precision of breeding and the return on investment. These findings on sika deer can serve as a reference model for genetic research on growth traits in other species and provide case support for cross-species comparative genetics. However, larger sample sizes and independent genomic datasets are required to confirm general applicability in future studies.

Most of the 12 body conformation traits in this study exhibited moderate to high heritability, indicating substantial genetic potential for improving body conformation in sika deer. Zhou et al. [30] reported that the heritability of body weight traits in sika deer was 0.62, which is higher than the heritability of body weight (0.38–0.47) estimated in our analysis. One reason for this discrepancy may be that the model overestimated dominance genetic variance, thus underestimating additive genetic variance and leading to a lower estimated heritability. Another reason may be that the kinship matrix constructed from whole-genome SNP information can more accurately reflect the genetic relationships between individuals when compared to that constructed from the pedigree [31]. In this study, the heritability of body conformation traits in 2-year-old sika deer was higher than the findings of Li et al. [32] on the growth traits of sika deer. This difference may be attributed to variations in breeding history, analytical methods, and the data structures. Williams et al. [28] reported that the heritability of body weight in 1.5-year-old white-tailed deer was 0.58–0.64, which is higher than the body weight heritability in this study (0.38–0.47). The possible reason is the breed differences and different breeding objectives, such as stronger artificial selection pressure in sika deer breeding.

### 4.2. Genetic Correlation Among Body Conformation Traits in Sika Deer

In our analysis of body traits of sika deer, a very strong positive genetic correlation was observed between BW, BH, BL, CC, and CD. This result is similar to that in the estimation of genetic parameters for the growth traits of Xinjiang cashmere goats, where strong positive genetic correlations were observed between body length and height, chest girth and height, and chest girth and body length [33]. For the cephalic traits, strong genetic correlations were observed between HL, FW, and HHD. Notably, a strong negative genetic correlation (−0.67 to −0.73) was found between head length (HL) and horn base distance (HHD), which likely arises from developmental trade-offs during cranial osteogenesis, where limited skeletal resources are differentially allocated to longitudinal head growth versus transverse horn base expansion [34]. This pattern may also reflect antagonistic pleiotropy, whereby shared genetic loci exert opposing regulatory effects on these two cranial morphological traits [35]. Similar negative genetic correlations among skeletal traits have been reported in cervid antler morphology, indicating widespread developmental constraints in the ungulate skull [36]. Meanwhile, strong positive genetic correlations were observed between HHGL and HHGR. Since TC and TL may be easily influenced by deer breeding management and external environmental conditions, these two traits in sika deer have a relatively low genetic correlation with other traits. The genetic correlations among body conformation traits indicate that selection for one trait can indirectly affect others and that these traits may share a common genetic basis, which can be used to establish more efficient breeding strategies for sika deer.

Some of the body conformation traits selected in this study, such as body length and chest girth, are easy to measure and exhibit a stably high genetic correlation with venison yield traits, and therefore can serve as efficient indirect selection indices. Their application may break through the technical bottlenecks associated with traditional slaughter-based measurements, facilitating the early, accurate, and low-cost selection of reserve sika deer individuals and providing key theoretical and technical support for the breeding of meat-type sika deer varieties.

### 4.3. Applicability and Advantages of Multi-Trait GWAS

Body conformation traits are typically regulated by multiple genes. By utilizing phenotypic correlations and integrating minor genetic effects, multi-trait GWAS can effectively improve statistical power and enhance the detection of novel significant SNP loci [37,38]. Using a similar strategy, Li et al. [16] conducted a multi-trait GWAS on body conformation traits in Chinese Holstein cattle and identified 93 new SNPs. Li et al. [32] performed multi-trait GWAS for sheep weaning and yearling weights, detecting 93 novel SNPs. Gao et al. [34] identified three novel SNPs associated with carcass weight, carcass length, and chest depth in Huaxi cattle. These findings indicate that for genetically correlated traits, multi-trait GWAS can serve as a useful supplement to single-trait GWAS and effectively improve its statistical power. We confirmed that some body conformation traits of sika deer were genetically correlated. It is worth noting that compared with 49 SNP identified by single-trait GWAS, a total of 114 novel SNPs were detected using multi-trait GWAS. (i.e., SNPs not detected in the single-trait analysis). The results indicated that it is both reasonable and necessary to combine single-trait GWAS and multi-trait GWAS, thereby improving the efficiency of SNP identification.

### 4.4. Age-Stratified GWAS and Comparative Analysis of Conformation Traits

Our comparative GWAS analyses across the 2-year-old, 3-year-old, and ≥4-year-old groups revealed pronounced age-dependent heterogeneity in the genetic architecture of conformation traits, consistent with previous findings in livestock species [39,40]. In single-trait analysis, the number of significant SNPs and candidate genes progressively declined with age, suggesting a stronger genetic influence on phenotypic variance during early growth (2 years old)—a period of rapid tissue proliferation and morphological differentiation [41]. As individuals mature to ≥4 years old, detectable genetic effects attenuate, potentially reflecting phenotypic stabilization or stabilizing selection [42].

In contrast, multi-trait GWAS identified the highest density of significant loci and candidate genes in the 3-year-old group, indicating this age as a critical developmental window where multiple conformation traits are shaped by a complex network of pleiotropic genes [43]. By leveraging genetic correlations among the 12 traits, the multi-trait model consistently outperformed the single-trait model across all age groups, uncovering more variants and genes and highlighting its advantage in capturing polygenic and pleiotropic architectures that remain masked in single-trait analyses [37,40].

Collectively, these findings demonstrate that the genetic basis of conformation traits evolves with development. Early growth is driven by large-effect, trait-specific loci, while mid-development (3 years old) is governed by a complex polygenic network. This age-dependent heterogeneity underscores the necessity of stage-specific modeling, as pooled analyses would obscure temporal genetic architecture and limit our understanding of trait formation [39,44].

Furthermore, genomic inflation factor (λ) was assessed across all single-trait and multi-trait GWAS performed in this study. Mild inflation was detected for partial phenotypic traits, which primarily originated from population stratification, hidden genetic relatedness among individuals, and the polygenic architecture of body conformation traits in sika deer. Fortunately, the linear mixed model implemented in GEMMA adequately eliminated these confounding biases. Most λ statistics were close to 1.0, indicating that the detected association signals were robust with low false-positive rates.

### 4.5. Candidate Genes and Pathways Identified for Facilitating Breeding of Sika Deer

Within the haplotype block at the 3.78–3.85 Mb region on chromosome 16, we detected two loci significantly associated with the BW, BH, and CD traits and identified two candidate genes, including *Slc25a37* and *NKX3.1*. Specifically, *Slc25a37* is a mitochondrial iron transporter whose main function is to transport cytoplasmic iron into mitochondria for hemoglobin synthesis and iron-sulfur cluster biosynthesis. It has been reported that *Slc25a37* knockout mice experience embryonic lethality due to mitochondrial iron deficiency, and erythroid-specific knockout leads to severe anemia and growth arrest [45,46], indicating that this gene is crucial for growth and development. Interestingly, *NKX3.1* (NK3 Homeobox 1) belongs to the NK homeobox transcription factor family, which may be involved in regulating prostate development and maintain homeostasis. It is a classic tumor suppressor gene for prostate cancer; loss of its expression is closely associated with tumor occurrence and progression and it may also serve as a pathological diagnostic marker [47,48]. While no studies have reported its direct correlation with body traits in livestock or poultry to date, its conserved biological functions suggest a potential role in regulating animal growth and development, providing a theoretical basis for investigating its effects on body size traits in sika deer.

Furthermore, our GO and KEGG enrichment results indicated that several signaling and functional pathways involving candidate genes may contribute to the formation of body conformation traits in sika deer. For example, the steroid hormone biosynthesis pathway, including *UGT2B4*, *UGT2B31*, *CYP21*, and *DD3*, is essential for hormonal regulation and metabolism. For another example, the drug metabolism–cytochrome P450 pathway, including *UGT2B4*, *UGT2B31*, *FMO1*, and *FMO4*, is essential for hormone homeostasis and oxidative stress regulation.

*UGT2B4* and *UGT2B31* are shared genes in two metabolic pathways, both of which belong to the UGT2B subfamily. Genetic polymorphisms of human UGT2B subtypes are closely associated with disease susceptibility and interindividual differences in drug metabolism [49,50]. Xing et al., [21] using a high-quality sika deer reference genome, confirmed that *UGT2B4* and *UGT2B31* are synchronously upregulated in the liver. By catalyzing the glucuronidation of toxic molecules, these two genes significantly increase the water solubility of the toxins, which are then excreted via bile or urine, thereby alleviating toxin-induced damage to the intestinal and hepatic tissues. On the one hand, we infer that these two genes may ensure the normal absorption of nutrients, thus providing essential raw materials for muscle protein synthesis and bone calcification. On the other hand, they may maintain the stability of core hepatic functions such as gluconeogenesis and lipid metabolism, ensuring that absorbed nutrients are prioritized for growth and development rather than for repairing toxin-induced tissue damage. This consequently improves feed conversion efficiency and growth rate, ultimately regulating body weight gain and body conformation development in sika deer.

*CYP21* encodes 21-hydroxylase, a member of the cytochrome P450 superfamily. This gene modulates the synthesis of glucocorticoids, mineralocorticoids, and androgens, thereby regulating cell proliferation, metabolism, and growth-related signaling pathways [51]. Studies in ruminants have demonstrated that the RFLP/HpaII polymorphism in the bovine *CYP21* promoter region is significantly associated with 450-day body weight, with T allele carriers exhibiting a faster growth rate [52]. In sheep, polymorphisms in the *CYP21* gene have been reported to be correlated with reproductive performance, which may indirectly affect maternal nutrient allocation and offspring growth [53]. These findings suggest that *CYP21* may regulate body size development in ruminants via the hormonal axis.

The *DD3* gene encodes dihydrodiol dehydrogenase 3, which belongs to the aldo-keto reductase (AKR) superfamily. Dihydrodiol dehydrogenase 3 exhibits dual activities as a dihydrodiol dehydrogenase and a prostaglandin F synthase. In ruminants, *DD3* may maintain growth homeostasis via detoxification metabolism and may regulate the hormonal axis through prostaglandin synthesis. It has also been reported to be associated with growth rate and production performance in cattle [54,55]. This gene is highly conserved among ruminants. Given the hormone-dependent characteristics of sika deer body conformation traits, it is speculated that *DD3* may be involved in the formation of sika deer body size traits by regulating skeleton and muscle development as well as energy metabolism. However, its specific function and molecular mechanism in sika deer remain to be further verified experimentally.

As for the tube circumference trait, although direct studies on *FMO1* and *FMO4* are limited, the FMO family, as a set of important flavin-containing monooxygenase genes, exerts core functions in the metabolism of exogenous compounds and the oxidative modification of endogenous signaling molecules, which are confirmed in *FMO2*, *FMO3*, and *FMO5* in various species. For example, research by Hosseini et al. [56] suggests that *FMO2* may be involved in controlling fat deposition in sheep tails by mediating adipogenesis and fatty acid biosynthesis. Some studies report that *FMO3* can influence energy homeostasis and body weight in mice by regulating bile acid metabolism [57]. Meng et al. [58] compared the adipose tissue transcriptomics data of fat-tailed and short-tailed sheep, identifying genes related to lipid metabolism (*FMO3*). In addition, a copy number variation in *FMO5* has been identified in European adult populations as being associated with height and body mass index [59].

### 4.6. Limitations and Future Studies

There are many successful GWAS studies on body conformation traits in ruminants, which provide pertinent reference for our study. Based on the genomic loci and candidate genes associated with body conformation traits in sika deer identified in our analysis, subsequent molecular functional validation of the identified candidate genes can potentially be carried out in the future. For instance, researchers may further investigate these genetic determinants through in vitro cell experiments, in vivo functional assays, and multi-omics integrative analyses. Furthermore, an independent validation cohort was not employed in the present study, which may limit the generalizability of the identified significant SNPs. Additionally, the relatively small sample size may have reduced statistical power for detecting loci with weak or moderate effects. While strict multiple testing corrections helped control false positives, they might also have excluded some biologically meaningful weak signals. These limitations suggest that future studies should use larger populations, multi-environment data, and advanced methods such as Bayesian or machine learning approaches. Resolving these issues will help clarify the genetic basis of body conformation traits in sika deer and support more precise genomic selection.

## 5. Conclusions

In this study, genetic parameter estimation and single-trait and multi-trait GWASs on 613 sika deer showed that most of the 12 body conformation traits had moderate to high heritability, with strong genetic correlations among related traits. Additionally, we identified 163 SNPs (196 candidate genes) and 17 genes detected by both GWAS methods. These results indicate that sika deer body conformation traits may be regulated by complex molecular networks involving candidate genes and two enriched KEGG pathways (steroid hormone biosynthesis, drug metabolism–cytochrome P450). The identified key SNPs and candidate genes could provide specific genetic markers for precise genomic selection and marker-assisted breeding of sika deer. Future work will validate key SNPs/genes, expand sample size, and integrate markers into breeding models to help accelerate the genetic improvement of sika deer.

## Figures and Tables

**Table 1:** Descriptive statistics of twelve body conformation traits of sika deer.

Traits	Group	N	Max	Min	Mean	SD	CV (%)
BW (kg)	1	254	130.00	67.00	96.74	13.03	13.47
	2	189	157.20	85.00	114.07	12.94	11.34
	3	322	179.00	86.00	129.47	16.97	13.11
BH (cm)	1	254	117.00	81.00	99.19	5.33	5.37
	2	189	120.00	91.00	101.25	4.75	4.69
	3	322	121.00	87.00	103.12	5.15	5.00
BL (cm)	1	254	125.50	80.00	103.83	6.32	6.08
	2	189	127.00	92.00	108.07	5.77	5.34
	3	322	129.00	90.00	111.00	6.35	5.72
CC (cm)	1	254	162.00	78.00	114.59	9.64	8.41
	2	189	160.00	95.00	118.99	10.19	8.57
	3	322	172.00	100.00	126.50	11.53	9.12
CD (cm)	1	254	59.00	38.00	45.20	3.41	7.55
	2	189	55.00	41.00	46.84	2.72	5.80
	3	322	78.00	42.00	49.71	3.73	7.51
TC (cm)	1	254	14.50	9.00	10.82	0.78	7.19
	2	189	17.00	9.00	11.19	0.87	7.81
	3	322	15.00	9.50	11.34	0.77	6.77
TL (cm)	1	254	23.00	10.00	15.97	2.28	14.26
	2	189	21.50	11.00	16.14	1.99	12.30
	3	322	21.50	11.00	15.96	2.06	12.88
HL (cm)	1	254	34.50	20.00	27.13	3.36	12.38
	2	189	35.00	21.00	29.09	2.21	7.60
	3	322	36.00	20.00	29.95	2.30	7.69
FW (cm)	1	254	16.00	10.00	12.81	1.50	11.70
	2	189	19.00	10.00	14.01	1.43	10.21
	3	322	19.00	10.50	14.74	1.72	11.66
HHD (cm)	1	254	18.00	4.50	9.94	1.44	14.66
	2	189	14.50	5.00	10.15	1.29	12.71
	3	322	17.00	3.50	10.64	1.43	13.40
HHGL (cm)	1	254	24.00	9.00	14.32	2.58	18.00
	2	189	25.00	11.20	15.86	2.61	16.43
	3	322	31.00	12.00	18.72	3.47	18.56
HHGR (cm)	1	254	24.00	9.00	14.30	2.58	18.07
	2	189	26.00	11.00	16.04	2.76	17.18
	3	322	36.50	12.00	18.72	3.39	18.11

Notes: body weight (BW), body height (BH), body length (BL), chest circumference (CC), chest depth (CD), Tube Circumference (TC), tail length (TL), head length (HL), forehead width (FW), horn handle distance (HHD), left horn handle girth (HHGL), and right horn handle girth (HHGR). Group 1: 2-year-olds, Group 2: 3-year-olds, and Group 3: ≥4-year-olds.

**Table 2:** Estimation of heritability of body conformation traits in three age groups of sika deer.

Traits	Group	σ_a_^2^	SE	σ_e_^2^	SE	h^2^ (95% CI)	SE
BW	1	64.89	58.36	106.44	53.86	0.38 (0.00–1.00)	0.32
	2	99.24	58.58	142.81	51.23	0.41 (0.12–0.70)	0.15
	3	133.37	58.42	150.77	48.96	0.47 (0.12–0.82)	0.18
BH	1	9.01	4.21	11.81	3.70	0.43 (0.06–0.80)	0.19
	2	9.38	4.31	18.17	4.41	0.34 (0.05–0.63)	0.15
	3	6.79	3.88	18.86	3.76	0.26 (0.00–0.55)	0.15
BL	1	10.05	6.06	22.39	5.70	0.31 (0.00–0.66)	0.18
	2	16.17	7.33	15.30	6.28	0.51 (0.10–0.92)	0.21
	3	16.47	5.74	21.25	4.99	0.44 (0.17–0.71)	0.14
CC	1	60.89	17.98	51.87	13.71	0.54 (0.19–0.89)	0.18
	2	83.41	18.54	115.19	12.81	0.42 (0.17–0.67)	0.13
	3	40.93	21.12	83.78	19.35	0.33 (0.02–0.64)	0.16
CD	1	3.62	1.47	4.61	1.23	0.44 (0.07–0.81)	0.19
	2	2.42	1.32	3.97	1.22	0.38 (0.01–0.75)	0.19
	3	3.01	1.06	5.49	1.42	0.35 (0.15–0.55)	0.10
TC	1	0.27	0.11	0.25	0.09	0.52 (0.15–0.89)	0.19
	2	0.28	0.09	0.27	0.08	0.50 (0.25–0.75)	0.13
	3	0.32	0.07	0.38	0.06	0.46 (0.19–0.73)	0.14
TL	1	1.31	0.79	4.15	0.80	0.24 (0.00–0.55)	0.16
	2	1.25	0.65	3.98	0.86	0.24 (0.00–0.53)	0.15
	3	2.06	0.87	6.09	1.45	0.25 (0.01–0.49)	0.12
HL	1	3.06	1.44	5.34	1.33	0.36 (0.05–0.67)	0.16
	2	1.23	0.79	2.53	0.75	0.33 (0.00–0.72)	0.20
	3	2.68	0.97	5.58	1.32	0.32 (0.07–0.57)	0.13
FW	1	0.36	0.29	1.88	0.32	0.16 (0.00–0.41)	0.13
	2	0.81	0.32	3.61	0.28	0.18 (0.00–0.51)	0.17
	3	0.39	0.20	1.14	0.20	0.25 (0.00–0.50)	0.13
HHD	1	0.32	0.25	1.22	0.25	0.21 (0.00–0.52)	0.16
	2	0.43	0.23	1.62	0.21	0.21 (0.00–0.60)	0.20
	3	0.22	0.22	1.48	0.24	0.13 (0.00–0.38)	0.13
HHGL	1	2.00	0.89	3.71	0.78	0.35 (0.00–0.72)	0.19
	2	0.98	0.69	2.14	0.58	0.31 (0.00–0.64)	0.17
	3	1.78	1.51	5.07	1.58	0.26 (0.00–0.53)	0.14
HHGR	1	2.66	0.97	5.92	0.78	0.31 (0.00–0.68)	0.19
	2	1.74	1.37	4.32	1.31	0.29 (0.00–0.72)	0.22
	3	3.82	1.69	6.41	1.52	0.37 (0.08–0.66)	0.15

**Table 3:** KEGG analysis of candidate genes related to body conformation traits.

Term	Description	Count	%	*P*. Adjust	Genes
ko00140	Steroid hormone biosynthesis	4	6.15	0.0468	*UGT2B4*, *UGT2B31*, *CYP21*, *DD3*
ko00982	Drug metabolism–cytochrome P450	4	6.15	0.0468	*UGT2B4*, *UGT2B31*, *FMO1*, *FMO4*

## Data Availability

The data that support the study findings are available from the authors upon request.

## Supplementary Materials

The following supporting information can be downloaded at: https://www.mdpi.com/article/10.3390/ani16091325/s1, Figure S1: SNP density map following quality control with the Sika Deer 100K SNP Liquid Chip. Figure S2: A trait frequency and distribution density line for body weight (a), body height (b), body length (c), Chest circumference (d), Chest depth (e), Tube circumference (f), Tail length (g), Head length (h), Forehead width (i), Horn handle distance (j), Left horn handle girth left (k), and Right horn handle girth(l); Figure S3: Linkage disequilibrium (LD) decay plot of the Sika deer population; Figure S4: A bubble diagram of KEGG pathways; Table S1: Significant loci and genes identified by single- and multi-trait GWAS; Table S2: Genomic inflation factor (λ) values for all single-trait and multi-trait GWAS analyses; Table S3: Number of significant SNPs and candidate genes detected by single- and multi-trait GWAS in three age groups.

## Abbreviations

GWAS	Genome-Wide Association Study
SNP	Single Nucleotide Polymorphism
BW	Body Weight
BH	Body Height
BL	Body Length
CC	Chest Circumference
CD	Chest Depth
TC	Tube Circumference
TL	Tail Length
HL	Head Length
FW	Forehead Width
HHD	Horn Handle Distance
HHGL	Left Horn Handle Girth
HHGR	Right Horn Handle Girth
PCA	Principal Component Analysis
HWE	Hardy–Weinberg Equilibrium
REML	Restricted Maximum Likelihood
LMM	linear mixed model
QQ	Quantile–quantile
SD	Standard Deviation
CV	Coefficient of Variation
GO	Gene Ontology
KEGG	Kyoto Encyclopedia of Genes and Genomes
