# Supplementary material for: Single- and Multi-Trait GWASs Combined with Genetic Parameter Estimation Reveal Candidate Genes for Body Conformation Traits in Sika Deer (Cervus nippon)
Source: Animals (Basel). 2026 Apr 27;16(9):1325. doi: 10.3390/ani16091325 (PMC13162871; doi:10.3390/ani16091325)
Supplement: Supplementary file 1 [file animals-16-01325-s001.zip › Supplement information.pdf]

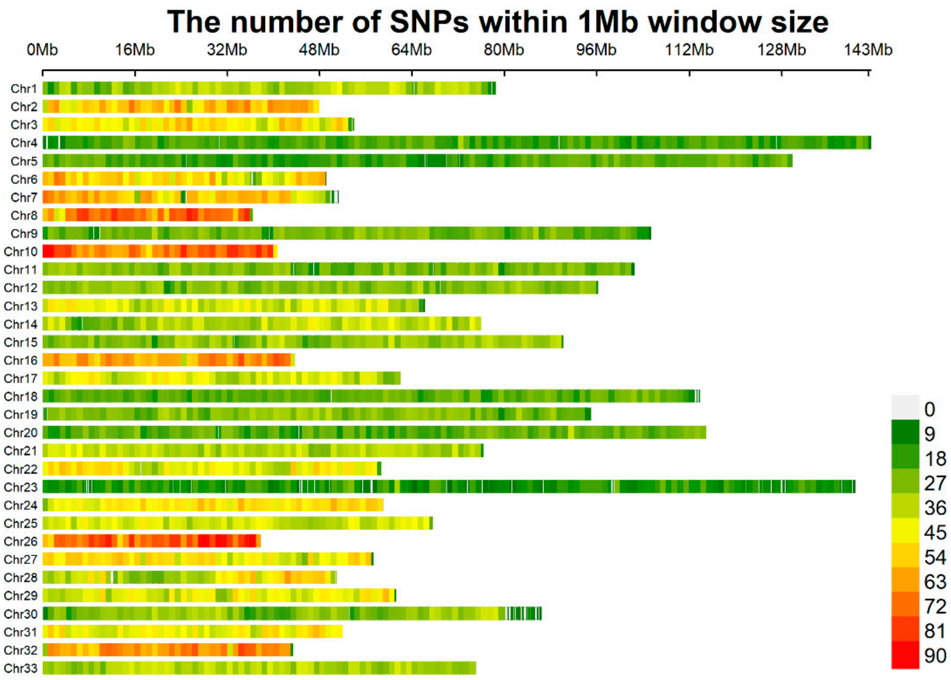

Figure S1 SNP density map following quality control with the sika deer 100K SNP Liquid Chip.

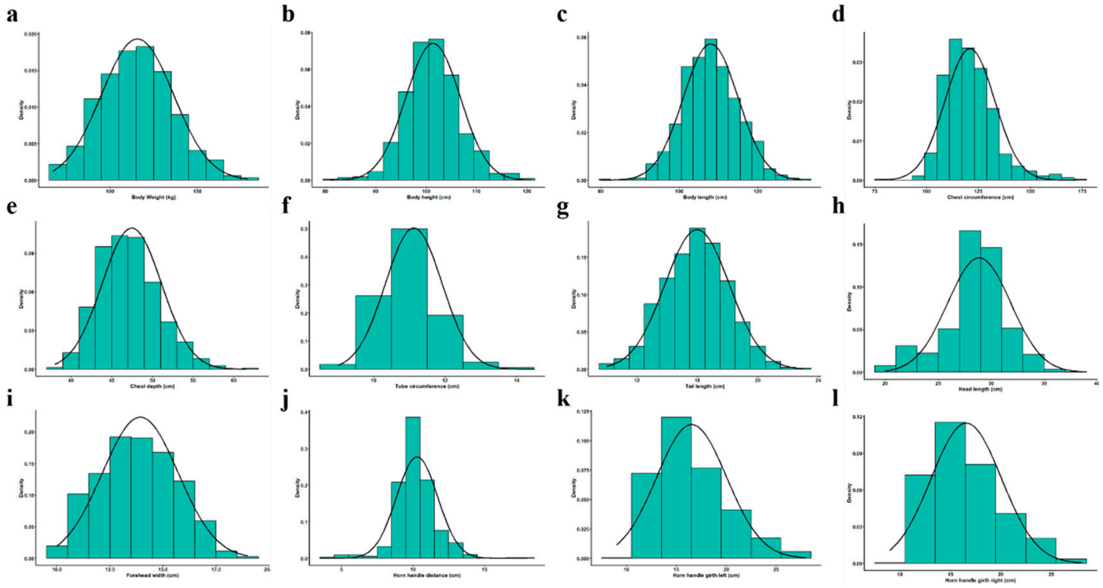

Figure S2 A trait frequency and distribution density line for Body weight (a), Body height (b), Body length (c), Chest circumference (d), Chest depth (e), Tube circumference (f), Tail length (g), Head length (h), Forehead width (i), Horn handle distance (j), Left horn handle girth (k), and Right horn handle girth (l).

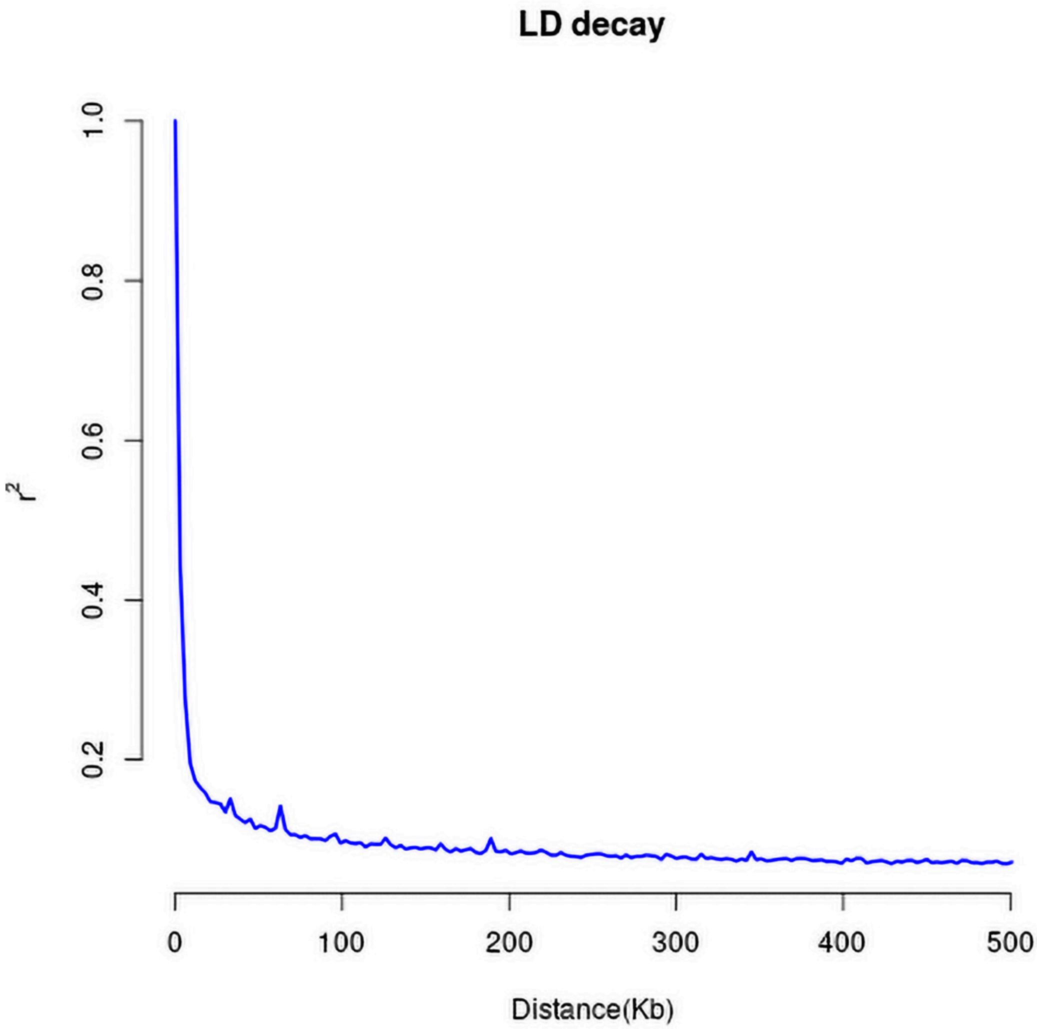

Figure S3. Linkage disequilibrium (LD) decay plot of the Sika deer population.

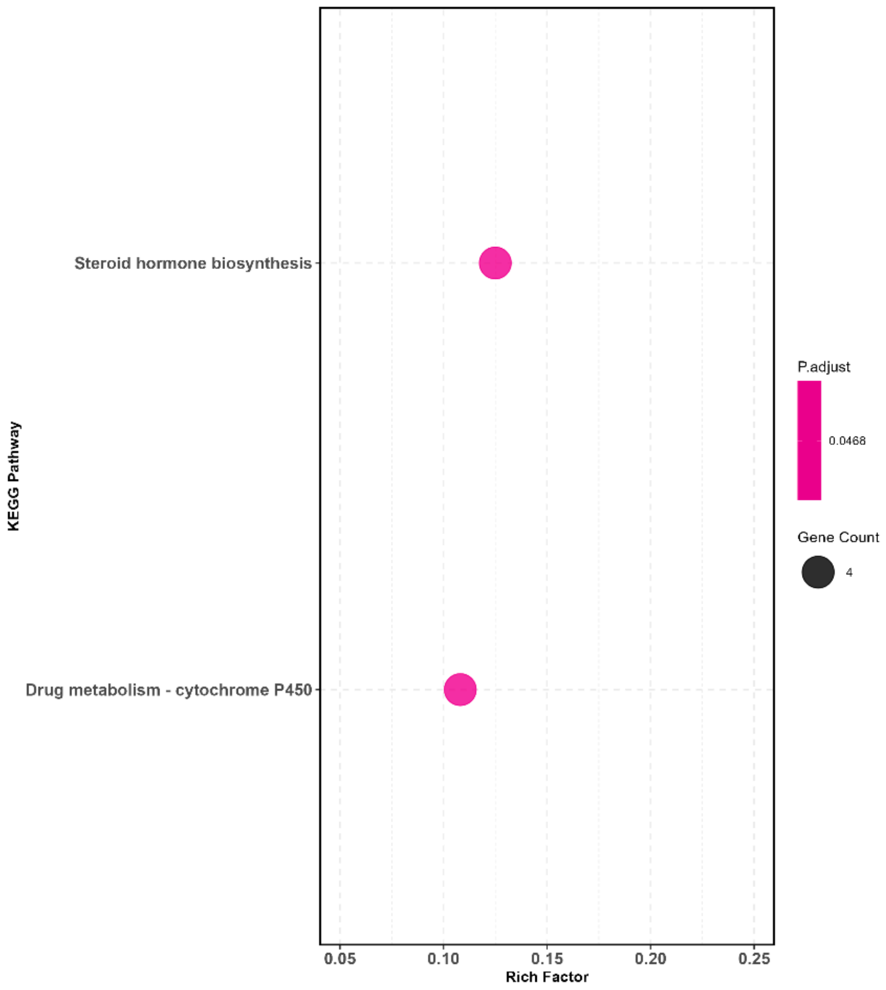

Figure S4 A bubble diagram of KEGG pathways.
